# Editorial: Is the Recent Burst of Therapeutic Anti-tumor Antibodies the Tip of an Iceberg?

**DOI:** 10.3389/fimmu.2018.00442

**Published:** 2018-03-05

**Authors:** Leonor Kremer, Jose A. Garcia-Sanz

**Affiliations:** ^1^Centro Nacional de Biotecnologia, Department of Immunology and Oncology, Spanish National Research Council (CSIC), Madrid, Spain; ^2^Cancer Genetics and Cancer Stem Cell Laboratory, Centro de Investigaciones Biologicas, Department of Molecular Biomedicine, Spanish National Research Council (CSIC), Madrid, Spain

**Keywords:** antitumor therapeutic antibodies, antibodies in combinations, effective cancer therapies, therapeutic antibodies, immunotherapy

**Editorial on the Research Topic**

**Is the Recent Burst of Therapeutic Anti-tumor Antibodies the Tip of an Iceberg?**

To summarize in a single research topic, all the useful information regarding the generation, characterization, and applications of antitumor therapeutic antibodies, including basic research, preclinical and clinical phases, turns out an impossible task. Therefore, our humble aim was to gather manuscripts from a group of researchers with expertise in the area, who through a set of 18 original research or review articles provide relevant and up-to-date information on several relevant aspects of this topic, exemplifying the ways in which antibody therapies will progress through technological advances in the generation of antibodies, the discovery of new targets, or new clinic applications that are being explored, as well as highlighting the main problems that remain to be solved.

The usefulness of antibodies for the treatment of infections, inflammatory diseases, and cancer cannot be questioned nowadays, and is evidenced by the large number of antibodies approved for their clinical use by the Food and Drug Administration (FDA) and the European Medicines Agency (EMA) (Corraliza-Gorjón et al.).

The first four articles of this research topic describe improved engineering approaches used to optimize antibody functions and the structural and functional advantages of different antibody formats. The review by Almagro and coworkers focuses in advances and problems on the design and development of antibodies for cancer therapy. It describes the methods used for the discovery, engineering, and optimization (for specific therapeutic uses) of FDA approved antibodies. It emphasizes on the development of antibodies with higher human content and less immunogenicity, compiling genetic engineering approaches for isotype switching and Fc fragment modifications allowing to modulate effector functions and bioavailability (half-life), as well as the technologies for Fv fragment engineering (Almagro et al.). Ilieva and coworkers describe on an original research article a novel approach to generate rapidly and with a high yield Fc mutant mAbs and ascertain their functional features. The approach includes coupling on a single expression vector antibody cloning together with simultaneous Fc region point mutagenesis and high-yield transient expression in human mammalian cells. The authors engineered antibody panels recognizing the cancer antigens HER2/neu and chondroitin sulfate proteoglycan 4 (CSPG4). Antibody variants with Fc mutations, affecting antibody-natural killer (NK) cell interactions, were generated in a few days from design to purified material. This strategy can facilitate the generation of antibodies with defined effector functions and potentially enhanced efficacy against tumor cells (Ilieva et al.). Bannas et al. comprehensively review the potential of nanobodies (Nbs) and Nb-based human heavy chain antibodies to overcome many of the limitations of the conventional, high molecular weight IgG antibodies. Nbs have small size, high solubility, high stability, and excellent tissue penetration *in vivo*. The authors discuss recent developments and perspectives for applications of Nb and Nb-based human heavy chain antibodies as antitumor drugs, describing methods and applications that use Nb linked genetically to other proteins or conjugated chemically at specific sites on other molecules to have additional properties. Iezzi et al. summarize how the unique properties of single-domain antibodies (sdAb), mainly their small size and high stability, make them suitable for image diagnostics and cancer treatment. Their contribution also describes the diversity of platforms available to generate new sdAb and some interesting examples of their therapeutic application.

Three contributions focus on the use of antibodies that target inhibitory immune checkpoints, an approach with demonstrated efficacy for the treatment of solid tumors (1–3). Aris et al. summarized clinical trials using immunomodulatory antibodies in combination with other strategies such as vaccines, in patients with cutaneous melanoma. Most of these studies include anti-CTLA-4 and anti-PD-1 mAbs and were designed aiming to obtain a durable antitumor immune response. The authors distinguish between immunotherapeutic strategies that “push” the tumor immunoreactivity from those which “release” inhibitory mechanisms of immune regulation. The original contribution from Taylor and Rudd focuses on the mechanisms of action of anti-PD-1 mAbs and their implications for immunotherapy. Their results demonstrate that glycogen synthase kinase 3 (GSK-3) plays a central role for priming CD8^+^ cytolytic T cells (CTL) in anti-PD-1 immunotherapy, as the inactivation of GSK-3 during priming can substitute for CD28 co-stimulation potentiating CTL function. Xu-Monette et al. discuss in their review the complexity of the PD-1–PD-L1 axis immunologic regulatory network, which can drive T cells to an irreversible dysfunctional state that cannot be rescued by blockade of this axis. The authors review the effectiveness of PD-1/PD-L1 blockade in many functional and clinical studies and the development of therapeutic strategies to overcome the resistance mechanisms, clearly improving our understanding on the mechanism(s) responsible for their efficacy.

Six contributions deal with the generation and use of antibodies for the treatment of hematological malignances either generating antibodies against new tumor targets or by selecting mAbs with improved functions against known targets. Marshall et al. extensively review the history of the development of clinically approved anti-CD20 antibodies, since rituximab, the first mAb approved by the FDA in 1997, until the new generation of anti-CD20 mAbs with enhanced effector functions. The authors discuss strategies to overcome mechanisms of resistance to the therapy and the use of combinations of anti-CD20 mAbs with other agents. Cuesta-Mateos et al. focus on antibodies evaluated in clinical trials for treating hematological malignancies, emphasizing on studies using mAbs directed against non lineage-specific antigens, including membrane surface glycoproteins, oncogenic receptors, chemokine receptors, antigens within the tumor niches, and immune checkpoint components. Schmitt et al. describe the use of a new generation of humanized antibodies targeting KIR3DL2, a member of the killer cell immunoglobulin-like receptor (KIR) family, for the treatment of cutaneous T-cell lymphomas, discussing aspects of KIR3DL2 on the functions of CD4^+^ T cells and highlighting preliminary clinical studies using the anti-KIR3DL2 mAb IPH4102 for the treatment of cutaneous T-cell lymphomas. The original contribution by Somovilla-Crespo et al. describes the generation and characterization of a novel anti-CCR9 mAb (92R), able to inhibit the growth of human CCR9^+^ leukemia in NSG mice xenografts. This chemokine receptor has been proposed as an attractive target for the treatment of human CCR9^+^ T cell acute lymphoblastic leukemia, since its normal expression is restricted to a low percentage of circulating immune cells. Ilieva et al. review the potential of antibodies directed to the Chondroitin Sulfate Proteoglycan 4 (CSPG4), overexpressed in several malignant diseases, with apparently restricted and low expression in normal tissues, and examine their direct antiproliferative/metastatic and immune activating mechanisms of action. Santamaria et al. discuss the possibilities of using therapeutic antibodies targeting the cancer stem cell (CSC) subpopulation, in particular since the CSC is responsible for tumor maintenance and metastasis generation.

Another group of contributions focuses on the advantages and challenges of treatments using combinations of antibodies with other therapeutic agents. Corraliza-Gorjón et al. summarize the use of antibodies in combinations with other biologicals for cancer treatment, describing their main characteristics, advantages, and challenges raised by the combinations. Muntasell et al. describe the main advances in the use of anti-HER2 antibodies in combination with harnessing NK cell responses for the treatment of HER2^+^ breast cancers. These approaches include the use of immune checkpoint blocking/stimulatory antibodies, cytokines, and toll-like receptor agonists.

Two contributions focus on how to design antibodies that can reach antigens or body sites that are normally inaccessible for these molecules. Trenevska et al. highlight that the target repertoire of actually approved antibodies is limited to tumor cell surface or soluble antigens. They describe how peptides from intracellular proteins that are presented on the cell surface in the context of major histocompatibility complex class I molecules can be targeted by antibodies known as T-cell receptor mimic or TCR-like antibodies. They summarize multiple approaches for targeting intracellular antigens, discussing their advantages and disadvantages and the potential to advance their therapeutic use into the clinic. Razpotnik et al. summarize new approaches to target brain tumors with therapeutic antibodies, especially malignant gliomas, as well as their potential drawbacks. They describe the properties of an antibody necessary to efficiently penetrate the blood–brain barrier; summarize studies demonstrating the successful brain delivery of single-chain fragment variable bispecific antibodies or cell-based systems, together with a summary of the most recent progress of related clinical trials.

The complexity of the use of therapeutic antibodies, alone or in combination keeps growing, as shown in the original research report from Marini et al. demonstrating the potential of using stable transfected human cells for the *in situ* expression of antibodies for cancer therapy. The authors generated a murine mesenchymal stem cell line stably secreting a dimeric EGFR-specific diabody single-chain TRAIL, demonstrating its therapeutic activity upon peritumoral injection in a Colo205 xenograft tumor model. These results support the potential of developing well-characterized stocks of stable drug-producing human MSC lines to establish standardized protocols of cell-based therapy broadly applicable in cancer treatments.

Most of the mAbs developed for tumor therapy are directed either against the tumor (tumor cell antigens, stromal cells, or secreted molecules) or against the host immune system (able to modify the immune response). These antibodies are used either alone, in combination, naked, as drug conjugates, as bivalent antibodies, or even as fusion proteins with cytokines/cytokine receptors. From our point of view, this line of research will expand generating new mAb, or Ab with non-conventional formats (including Nbs, multipecific Ab, etc.) against new tumor antigens. In addition, we believe that there will be a burst on the use of combinations of immunomodulatory antibodies with either mAbs against the tumor or with tumor-derived antigens administered as vaccines (in multiple forms, i.e., tumor cell extracts, plasmids, virus, peptides, or purified proteins). The aim in both cases is to overcome the mere usage of mAbs as passive immunotherapy and use them for active immunotherapies, either to restore the effector activity of the immune system or to generate a new immune response against non-self antigens (tumor antigens), bypassing the hijacking of the immune system by the tumor. The other area that will grow on the near future will be the use of mAbs combined with adoptive cell therapies (harnessed T- or NK-cells, cells carrying expression vectors allowing them to express antibodies, tumor antigens, or chimeric antigen receptor T cells).

These contributions summarize the recent progress on therapeutic use of antitumor antibodies, providing new and important information for the understanding of how cancer treatments using antibodies might evolve, highlighting many unresolved issues and controversies. Taken together, these contributions should allow to gather a representative view of the enormous potential of antibodies and antibody-based molecules as effective tools for cancer immunotherapy. We hope that this information will be useful for a wide audience, including researchers, immunologists, oncologist, and students of biomedical sciences.

## Author Contributions

LK and JG-S designed and wrote this editorial article.

## Conflict of Interest Statement

The authors declare that the research was conducted in the absence of any commercial or financial relationships that could be construed as a potential conflict of interest.
